# Neurodynamical model of confidence decision-making in LIP

**DOI:** 10.1186/1471-2202-12-S1-P65

**Published:** 2011-07-18

**Authors:** Andrea Insabato, Mario Pannunzi, Gustavo Deco

**Affiliations:** 1 Center for Brain and Cognition, University Pompeu Fabra, Barcelona, Spain; 2 ICREA, Barcelona, Spain

## 

Decision confidence, the degree of certainty to which a subject believes his choice is correct, is an emerging subject in neuroscience. On one hand it is a fundamental component of our subjective conscious experience. On the other hand it is crucial to many cognitive functions like action planning and learning. In the past the speed accuracy trade-off in decision-making has received much attention by the scientific community since it is a key aspect of simple decisions, used in experimental conditions. In the context of complex more “real” environments, where different strategies can be applied, besides speed and accuracy, *flexibility* also become very important. Flexibility is the ability of an agent to explore and use alternative strategies in order to reach the goals. In a more simple view flexibility is the ability of taking into account more options in a decision process. The selection between alternative strategies can be regulated by evaluating the confidence in a choice. If the confidence is too low and other strategies are available, then the subject will try an alternative. Therefore evaluating the confidence in a choice can be particularly helpful for agents in complex dynamic environments.

There is only few evidence about neural mechanisms underlying decision confidence [1][2]. New neurophysiological evidence about decision confidence comes from a recent study [3], that tested the ability of monkeys to choose, in a two choice random dot motion (RDM) task, between standard targets and a “sure” target appearing later. This target represents a small but certain reward. Recordings from LIP neurons during the behavioral task show a dependence of firing rate upon certainty in the decision.

We propose a theory about neuronal mechanisms of flexibility that can account for LIP data. The theory is implemented in a network model composed of integrate-and-fire neurons with biologically detailed synapses, including AMPA, NDMA and GABA receptors [4].

Our proposal is that, as reported by [2], the confidence is implicitly encoded in the firing-rate of the decision neurons, but there is no need for a reading out of this information. In our model the RDM stimulus generates a competition between two pools of decision neurons, a third pool remains silent until it is turned on by the third target presentation. When all three pools receive task salient stimulus they compete until a decision is reached. In this way the information about confidence in the first decision, stored in the firing-rate, is directly used into the network without the need of an other reading network [2].

We analyze the property of the network in a reduced mean-field model. The first bifurcation in the space of input is connected with speed accuracy trade-off [5]. Our finding is that the proximity with a second bifurcation enhances flexibility, raising the probability of alternative strategies.
